# Miniature Diamond-Based Fiber Optic Pressure Sensor with Dual Polymer-Ceramic Adhesives

**DOI:** 10.3390/s19092202

**Published:** 2019-05-13

**Authors:** Hyungdae Bae, Ayush Giri, Oluwafikunwa Kolawole, Amin Azimi, Aaron Jackson, Gary Harris

**Affiliations:** 1 The Department of Mechanical Engineering, Howard University, Washington, DC 20059, USA; ayush.giri@bison.howard.edu (A.G.); oluwafikunwa.kolawo@bison.howard.edu (O.K.); amin.azimi@howard.edu (A.A.); 2 Howard Nanoscale Science and Engineering Facility, Department of Electrical and Computer Science, NSF STC Center for Integrated Quantum Materials, Howard University, Washington, DC 20059, USA; aaron.jackson@howard.edu (A.J.); gharris@howard.edu (G.H.)

**Keywords:** fiber optic sensor, Fabry–Perot, micro-optical device, polycrystalline diamond

## Abstract

Diamond is a good candidate for harsh environment sensing due to its high melting temperature, Young’s modulus, and thermal conductivity. A sensor made of diamond will be even more promising when combined with some advantages of optical sensing (i.e., EMI inertness, high temperature operation, and miniaturization). We present a miniature diamond-based fiber optic pressure sensor fabricated using dual polymer-ceramic adhesives. The UV curable polymer and the heat-curing ceramic adhesive are employed for easy and reliable optical fiber mounting. The usage of the two different adhesives considerably improves the manufacturability and linearity of the sensor, while significantly decreasing the error from the temperature cross-sensitivity. Experimental study shows that the sensor exhibits good linearity over a pressure range of 2.0–9.5 psi with a sensitivity of 18.5 nm/psi (R^2^ = 0.9979). Around 275 °C of working temperature was achieved by using polymer/ceramic dual adhesives. The sensor can benefit many fronts that require miniature, low-cost, and high-accuracy sensors including biomedical and industrial applications. With an added antioxidation layer on the diamond diaphragm, the sensor can also be applied for harsh environment applications due to the high melting temperature and Young’s modulus of the material.

## 1. Introduction

Interest in the use of miniature fiber-optic pressure sensors for medical and industrial applications has progressively increased over recent decades. The increased interest is due to their unique attributes such as ultra-fast dynamic response, micro-scale size, high sensitivity, immunity to electromagnetic interference, and the convenience of light transmission/detection through optical fibers [1,2]. The compact dimensions of the fiber-optic sensors significantly improve the spatial resolution of measurements and, in the case of medical applications, patients’ comfort level. The various types of miniature optic sensors reported in the literature are based on the Fabry–Perot optical cavity [3,4,5,6,7,8,9,10,11,12,13,14,15,16]. An extrinsic Fabry–Perot (FP) cavity is formed at the tip of an optical fiber by using the end of the optical fiber surface and a reflective miniature diaphragm built on a support structure. The diaphragm deflects in response to variations of ambient pressure and causes changes in the interference signal generated by the FP cavity. Mainly, there are two different categories of materials for fiber optic sensors that make use of extrinsic FP cavity structure. The first category is ceramic materials (e.g., fused silica, silicon, zirconia, etc.) [4,11,17,18,19,20]. These materials are suitable for high-pressure and -temperature application due to the materials’ high mechanical strength and temperature stability. Temperature sensitivities of the materials are relatively low, which give those sensors low-temperature cross-sensitivities. The second category of materials is metals, polymers, and metal-polymer composites (e.g., silver, aluminum, UV curable polymers, BoPET (biaxially-oriented polyethylene terephthalate), etc.) [7,15,21,22,23,24]. It is more advantageous to achieve high-pressure sensitivity with these materials compared to ceramic counterparts due to the materials’ relatively low Young’s moduli. However, the sensors made of metal or polymers suffer from relatively high-temperature cross-sensitivity because of the high thermal expansion of the materials. If the sensors are used in a temperature changing environment, an additional temperature sensing and compensation schemes are required to compensate for the temperature cross-sensitivity of the sensors, and the operation temperature is relatively low due to the oxidation of metals or softening/burning of polymers (~200 °C).

**Figure 1:**
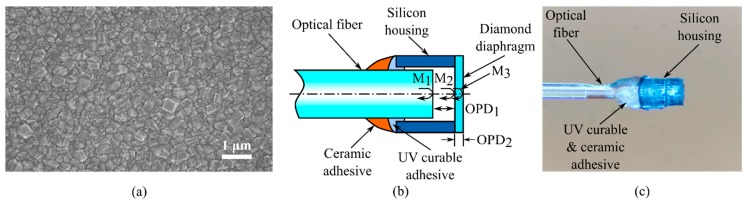
(**a**) A scanning electron micrograph of chemical vapor deposition (CVD) diamond film and (**b**) schematic of the diamond-based pressure sensor with a polymer-ceramic hybrid adhesive; (**c**) microscopic image of a fabricated sensor.

Polycrystalline diamond can be grown by chemical vapor deposition (CVD) and has many unique properties which can be exploited as a sensor material for various sensing applications. A scanning electron micrograph of CVD diamond film is shown in Figure 1a. CVD polycrystalline diamond films have outstanding properties of high Young’s modulus (1143 GPa) [25], low thermal coefficient of expansion (1–1.5 µε/°C), high melting temperature (above 1700 °C in vacuum or oxygen-free environment), ultra-high thermal conductivity (2200 W/cm·K) [26], and inertness to most acids and bases. The refractive index of diamond is 2.4168 (at 587.6 nm) and optically transparent between the deep ultra-violate and the infrared ranges [27]. Although there have been other types of pressure sensors based on diamond piezoresistors [28,29,30,31,32], there is only a limited number of works on optical fiber pressure sensor made of the diamond diaphragm [33]. In this work, a fiber optic sensor fabrication method was used, which makes use of dual polymer-ceramic adhesive for low-cost fabrication and relatively high operation temperature (~275 °C).

This study aims to design and fabricate a novel and miniature optical pressure sensor based on a polycrystalline diamond diaphragm. Figure 1b illustrates the schematic of the proposed pressure sensor, which consists of a cleaved or polished optical fiber, a silicon cavity structure, a UV curable adhesive, ceramic adhesive, and a synthetic polycrystalline diamond pressure sensing diaphragm. The UV curable adhesive was used for instant curing and sealing of the air cavity. The ceramic adhesive was added on top of the UV curable adhesive for improved linearity of the sensor signal and reduced variations in the pressure sensitivity. A microscopic image of a fabricated sensor is shown in Figure 1c. For high temperature application, an anti-oxidation layer such as titanium oxide (TiO_2_) or aluminum oxide (Al_2_O_3_) can be added to protect the diamond diaphragm from oxidation. The diamond melts at very high temperatures (above 1700 °C), but it suffers from oxidation in an oxygen-rich environment above 800 °C [34]. Exceptional thermal conductivity can help minimize thermal stress in the material and the time constant of temperature measurement. However, high-temperature sensing of the diamond-based pressure sensor is not within the scope of this work. The fabrication process of the proposed sensor is cost-efficient and can provide a good device-to-device uniformity since the diamond diaphragm and the optical housing fabrication processes are performed in batch with very good thickness and dimension control by using conventional semiconductor processes (e.g., CVD, photolithography, and deep reactive ion etching). The rest of fabrication processes can be either easily automated by using motorized precision stages with an optical vision system or performed in batch. Therefore, sensor fabrication can be cost-effective and suitable for low-cost applications. The sensor fabrication processes will be discussed in detail in the following section.

## 2. Sensor Design and Fabrication

The inner diameter of the optical cavity was chosen to be 135 µm considering the outer diameter of the optical fiber (i.e., 125 µm), the cavity etching tolerance, and required tolerance for fiber assembly. The thickness of the diamond layer was carefully designed to meet the designed pressure sensitivity and maximum pressure range while ensuring a linear sensor response. The thickness of the diamond diaphragm was designed to give a deflection of 10 nm/psi or higher and to operate at a pressure of 30 psi or higher using analytical solutions [22]. The analytical solution was verified based on the finite element method based on COMSOL Multiphysics. Based on the design processes, the thickness of the diamond diaphragm was selected to be 1.2 µm to achieve a pressure sensitivity of 14.3 nm/psi and a maximum pressure range of 33.79 psi.

**Figure 2:**
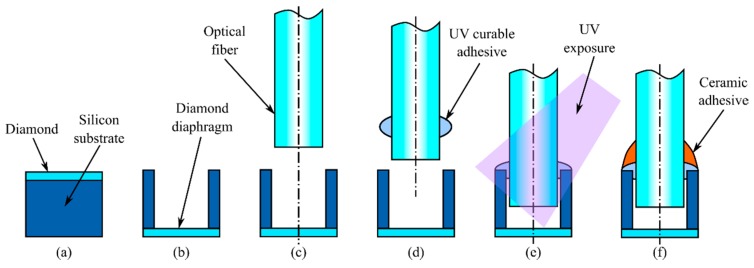
The fabrication process of the diamond-based pressure sensor (**a**–**f**).

Sensor fabrication consists of three steps that include: i) Growth of the diamond diaphragm on a silicon wafer, ii) fabrication of the optical housing structure, and iii) optical fiber alignment and mounting. The detailed fabrication processes are demonstrated in Figure 2. The first step is to grow the diamond layer on a silicon wafer. The 1.2 μm thick heteroepitaxial diamond film was grown in a hot filament chemical vapor deposition (HFCVD) system on single side polished p-type silicon wafers (Figure 2a). After cleaning and then removing the surface oxide, the Si wafers were sonicated in a diamond nano-particle slurry to embed diamond seed particles on the surface. The average crystal size in the diamond slurry we used is 5 nm. The diamond film was grown using hydrogen and methane as the source gases. During growth, the silicon wafer was maintained at 800 °C. This elevated temperature can generate residual stress in the deposited diamond film when the fabricated sensor is used at ambient temperature. The pressure sensitivity of the sensor can be precisely tuned in this step according to the application requirements. Optionally, the diamond layer can be patterned into individual circular islands using photolithography and reactive ion etching processes to define each pressure-sensing diaphragm. Secondly, the backside of the silicon wafer is patterned and etched using deep reactive ion etching (DRIE) (Figure 2b). Each Si housing structure is created by etching through the entire 350 μm thickness of the Si wafer. The diamond layer on the front side of the silicon wafer acts as an etch stop because of the large etch ratio difference between silicon and diamond layer. Lastly, an optical fiber is inserted into the Si housing defined by the DRIE process forming an FP optical cavity (Figure 2c–e). A single mode optical fiber with a diameter of 125 μm (SMF-28 Ultra, Corning, Corning, NY, USA) is first cleaved and cleaned to ensure particle free condition before the assembly. Then, the cavity inlet and the fiber are aligned using manual/piezo stages under microscopes. The alignment setup is comprised of two five-axis high-precision manual stages with attached piezo stages and two optical microscopes with CCD cameras positioned with 90° angle separation. Next, the optical fiber is carefully inserted into the housing structure while monitoring the gap distance between the cleaved fiber end the diamond diaphragm surface. Monitoring is performed by using the same optical system which will be used for the fabricated sensor interrogation. The optical interrogation system will be discussed in the following section in detail. The cavity length can be precisely measured and controlled with a resolution of less than 1 nm by using the optical interrogation system. Horizontal position and tilt alignments are ensured by the clearance between the Si housing and the inserted optical fiber. When the desired gap distance between the optical fiber and the diamond diaphragm is obtained, a small drop of UV curable adhesive (OP-5-20632, Dymax, Torrington, CT, USA) is applied between the fiber and silicon cavity inlet to fix the fiber and seal the formed optical cavity. Due to the capillary effect, the gap between the cavity wall and the optical fiber is filled. The UV light from a spot light source (LC8, Hamamatsu, Shizuoka, Japan) is then exposed to the applied UV curable polymer, securing the optical fiber to the cavity and sealing the air cavity at the vicinity of the end of the optical fiber (Figure 2e). To minimize shrinkage of the UV curable polymer a low-intensity exposure (10% of the full intensity for 30 s) is applied followed by a high-intensity exposure (100% of the full intensity for 60 s). The structure which holds the tube-shape silicon housing structure is then broken off by applying minimal force to the silicon structure which loosely holds the housing structure. Additional ceramic adhesive (618-N-VFG, Aremco, Valley Cottage, NY, USA) is applied on top of the cross-linked UV curable polymer and thermally cured after 4 h of air drying (Figure 2f). Thermal curing was performed at 150 °C and 300 °C for 2 h at each temperature. The addition of ceramic adhesive significantly improves the linearity of the pressure and temperature response by minimizing the viscoelastic behavior of the UV curable polymer.

## 3. Optical Interrogation and Signal Processing

**Figure 3:**
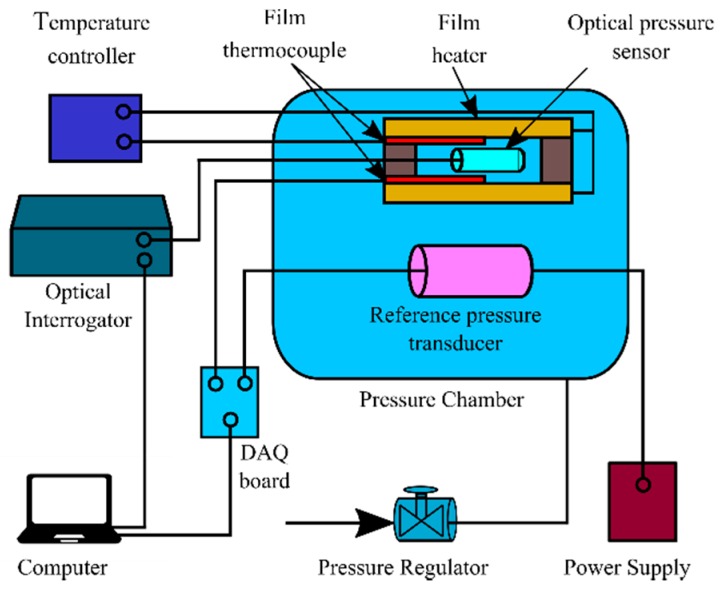
Experiment arrangement for pressure and temperature calibration.

The sensor was connected to a broadband optical interrogation system, which is composed of a 3 dB coupler (50:50 coupling ratio at λ = 780 nm, Thorlabs, Newton, NJ, USA), a broadband spectrometer (wavelength range: 697–971 nm, flame-T, Ocean Optics, Largo, FL, USA) 0.4 nm wavelength resolution, and a broadband light source (HL-2000-HP, Ocean Optics, Largo, FL, USA). Theoretically, SMF-28 Ultra used for sensors fabrication operates in single-mode in the wavelength range of the spectrometer. However, the length of the fiber for sensors fabrication is relatively short (~30 cm) so the multi-mode effect is not significant. The minor multi-mode effect is filtered during the signal processing using a low-pass filter. The spectrum position and the output of the reference sensor were collected by custom data acquisition code based on LabVIEW (National Instruments, Austin, TX, USA) while the chamber pressure and temperature were changed independently using a pressure regulator (Type 10, Bellofram Corp., Newell, WV, USA) and temperature controller (CN77332, Omega Engineering, Norwalk, CT, USA) with a thermocouple (CO1-K, Omega Engineering, Norwalk, CT, USA) and two heaters (KH-103/10, Omega Engineering, Norwalk, CT, USA). The experimental arrangement for pressure and temperature calibration is illustrated in Figure 3. A detailed description of the calibration setup can be found in one of the authors’ previous publications [35]. 

**Figure 4:**
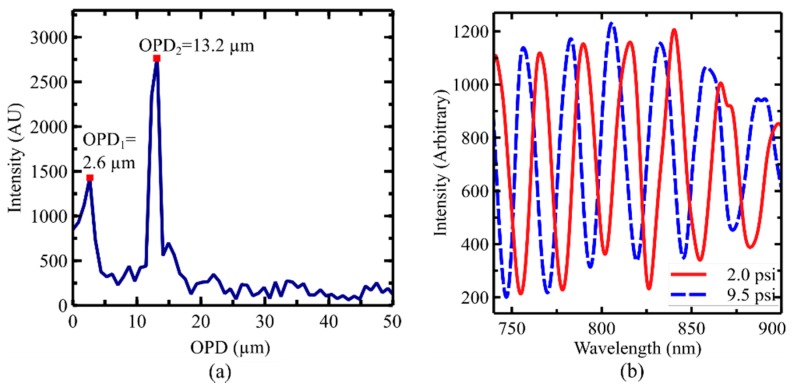
(**a**) Experiment arrangement for pressure and temperature calibration; (**b**) fast Fourier transform (FFT) result from the wavenumber spectrum of the sensor.

Three optical cavity cavities are generated by three distinctive optical interfaces (i.e., M_1_, M_2_, and M_3_ in Figure 1b) of the proposed sensor. The intensity profile of interference as a function of wavelength generated by the three cavities can be described as below [18]:

(1) $$\begin{array}{lll} I(\lambda ) & = & {\left|A_{1}-A_{2}\exp\left(-\frac{j4\pi (n_{\mathrm{air}}L_{\mathrm{air}})}{\lambda }\right)+A_{3}\exp\left(-\frac{j4\pi (n_{\mathrm{air}}L_{\mathrm{air}}+n_{\mathrm{diamond}}L_{\mathrm{diamond}})}{\lambda }\right)\right|}^{2}\\ & = & A_{1}^{2}+A_{1}^{2}+A_{1}^{2}-2A_{1}A_{2}\cos(\frac{n_{\mathrm{air}}L_{\mathrm{air}}}{\lambda })-2A_{2}A_{3}\cos(\frac{n_{\mathrm{diamond}}L_{\mathrm{diamond}}}{\lambda })\\ & & +2A_{3}A_{1}\cos(\frac{n_{\mathrm{air}}L_{\mathrm{air}}+n_{\mathrm{diamond}}L_{\mathrm{diamond}}}{\lambda }), \end{array}$$

where *A*_1_, *A*_2_, and *A*_3_ are the amplitude of the reflected electric fields from the end of the optical fiber (M_1_), the first diamond interface (M_2_), and the second diamond interface (M_3_), respectively, *n*_air_ is the refractive index of air, *L*_air_ is the length of the air cavity, *n*_diamond_ is the refractive index of diamond, *L*_diamond_ is the thinness of the diamond diaphragm, and *λ* is the free-space wavelength. As the ambient pressure increases the sealed air cavity length (*L*_air_) decreases due to the pressure difference between the two area which will result in a blue shift of the frequency generated by the air cavity. The diamond layer thickness (*L*_diamond_) can be considered constant since the stiffness of the solid diamond layer is much larger than that of the air cavity. To monitor the change of air cavity length with a high resolution, the optical frequency isolation and the one peak tracing were used in this work [36]. At first, the measured spectrum was converted to the wavenumber domain for optical frequency-based filtering. The converted wavenumber domain spectrum was interpolated to make it evenly spaced and resampled to reduce the wavenumber step size for a better resolution processing. The fast Fourier transform was applied to the spectrum to find the optical frequencies representing individual optical cavities in the sensor. Figure 4a shows a representation of the fast Fourier transform (FFT) result of the sensor wavelength spectrum. OPD_1_ (optical path difference) is from the optical cavity defined by the two interfaces of the diamond diaphragm. OPD_2_ is from the air cavity formed by the optical fiber end face and one of the diamond diaphragm interfaces (see Figure 1b). The optical cavity generated by the combination of the two cavities was relatively weak visibility of the interference signal. There is a relationship between the measured optical path difference (*OPD*) and the optical frequency (*f*) in the wavenumber domain which can be described as below:

(2) OPD=2nL=f,

where *n* is the refractive index of the optical cavity medium and *L* is the optical cavity length. Due to the limited wavelength range of the used spectrometer, the cavity length measurement based on the FFT is not high enough (~0.5 µm). Therefore, the found optical frequency from the FFT was only used as the center frequency for the following band-pass filtering of the spectrum using the Butterworth filter. The filter wavenumber spectrum was converted back to the wavelength domain and one peak tracing method was used to monitor the change of cavity length with high resolution [36]. Representative spectra from the tested sensor at two different pressures (2.0 and 9.5 psi) are shown in Figure 4b. The detailed signal processing method can also be found in one of the authors’ previous works [35].

## 4. Discussion: Sensor Calibration and Testing

**Figure 5:**
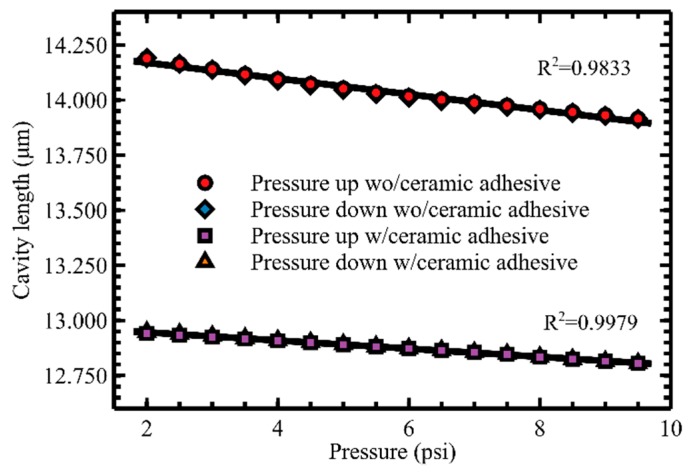
Pressure calibration curves for the sensor with only UV curable adhesive vs. with UV curable and ceramic adhesive at 24.5 °C.

Pressure calibration of the sensor was conducted in a pressure chamber with a reference pressure sensor (MMG250V10, Omega Engineering Inc., Norwalk, CT, USA) to quantify the changes in the sensor air cavity length with respect to the pressure changes. The calibration was performed in a pressure range of 2 to 9.5 psi. However, the upper limit of the pressure range was chosen to be 9.5 psi considering the limitation of the pressure chamber used for the calibration. The calibration result is shown in Figure 5. Two different sets of calibration data overlap closely between pressure increase and decrease cases showing relatively low hysteresis of the sensor. The calibration data show good linearity with an R^2^ value of 0.9979 and sensitivity of 18.5 nm/psi for combined data from increasing and decreasing pressure with a step size of 0.5 psi at room temperature of 24.5 °C. The sensitivity number is a little higher than that of the FEM model (i.e., 14.3 nm/psi) even though the residual stress in the diamond film reduce the sensitivity. It is believed that the sensitivity higher than the estimation is attributed to the pressure sensitivity coming from the deformation of UV adhesive under pressure change. The pressure resolution of the FP pressure sensor was determined to be 0.0075 psi by using the RMS error divided by the measured pressure sensitivity of the sensor. The pressure calibration result from a sensor before adding the ceramic adhesive is shown in the same plot of Figure 4 in red dots and blue diamonds. The same pressure range, step size, and sequence were used for both datasets. As shown in the result, R^2^ improved by 1.5% and the pressure sensitivity decreased by 48% after applying the ceramic adhesive. It is believed that the applied ceramic adhesive significantly reduced the deformation and viscoelastic behavior of the UV curable adhesive due to its high Young’s modulus compared to that of the UV curable adhesive. Because of the UV curable polymer shrinkage during the thermal curing of the ceramic adhesive, 8.3% cavity length shrinkage was also observed.

**Figure 6:**
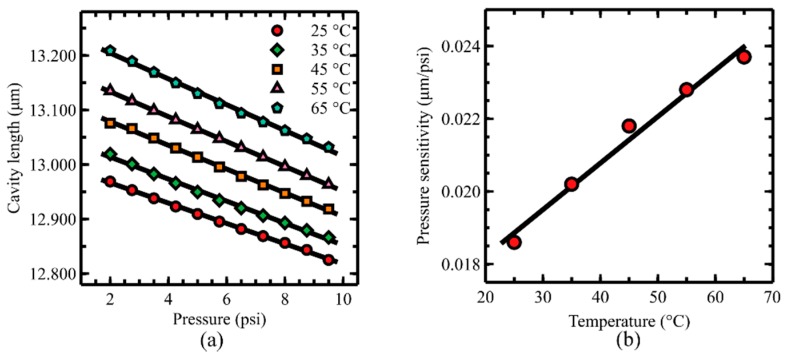
(**a**) Pressure calibration curves at five different temperature and (**b**) a temperature calibration curve at 2 psi.

Pressure calibrations were performed at five different temperatures from 25 to 65 °C with 0.75 psi step size (Figure 6a). The pressure sensitivity of the sensor was noted to increase from 18.6 to 23.7 nm/psi when the temperature increased from 25 to 65 °C. The increase in the pressure sensitivity with the increase in temperature is believed to come from softening of the UV curable adhesive in the sensor that was used with ceramic adhesive. A sensor without the ceramic adhesive shows 4.3 times larger sensitivity variation than that of a sensor with the ceramic adhesive in the same temperature range (i.e., from 25 to 65 °C). The pressure sensitivity change with respect to the temperature change is shown in Figure 6b.

**Figure 7:**
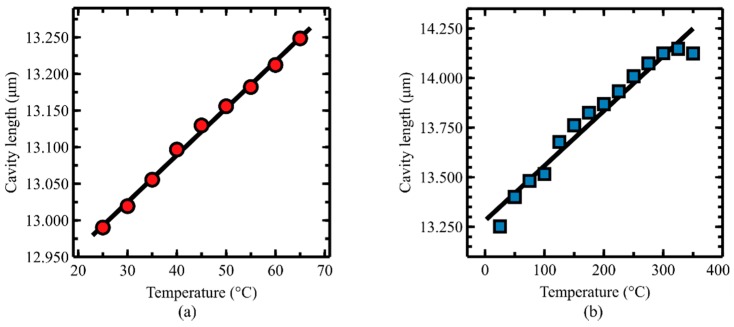
A temperature calibration curve (**a**) at 2 psi with a range of 25 to 65 °C and (**b**) at atmospheric pressure with a range of 25 to 325 °C.

To evaluate the temperature sensitivity of the sensor, a temperature calibration of the air cavity was performed. To measure the temperature sensitivity, the sensor was heated from 25 to 65 °C with an increment of 5 °C under the constant pressure of 2 psi. The cavity lengths were recorded at each temperature level. The obtained temperature calibration results are shown in Figure 7a. According to the result, a linear relationship between the air cavity length and temperature can be observed with good linearity (R^2^ = 0.9965) and a sensitivity of about 6.4 nm/°C. The temperature sensitivity of the sensor is coming from the expansions of the silicon housing, air trapped in the cavity, and the polymer-ceramic adhesive. Thermal expansion model of the sensor was not performed due to the large shape variation of the applied polymer-ceramic adhesive. The expansion of the polymer is believed to play a significant role in the overall thermal expansion because of its large coefficient of thermal expansion (45 µε/°C) compared to the rest of the materials [37]. The change of the cavity length due to temperature change can be compensated by adding another Fabry–Perot sensor or other types of temperature sensors (e.g., fiber Bragg grating) [35,38].

To investigate the maximum operating temperature of the sensor, an additional temperature calibration was performed with a larger temperature range than the previous temperature calibration. For the calibration, the sensor was heated from 25 to 325 °C with an increment of 25 °C under the atmospheric pressure. The cavity lengths were recorded at each temperature level. The obtained temperature calibration results are shown in Figure 7b. According to the result, a relatively linear relationship between the air cavity length and temperature is observed up to 275 °C which is believed to be the maximum operating temperature of the sensor. This operating temperature is much higher than the glass transition temperature of the applied UV adhesive for sensor fabrication (glass transition temperature of the UV adhesive: 78 °C). The ceramic adhesive applied on top of the UV adhesive could have increased the operating temperature of the sensor to a temperature that is much higher than the glass transition temperature of the UV adhesive (operation temperature of the ceramic adhesive: 1760 °C). The increased manufacturability and the operating temperature of the sensor due to the polymer/ceramic adhesive is one of the advantages of the proposed sensor fabrication method.

## 5. Conclusions

In this article, a miniature diamond-based fiber optic sensor fabricated using a silicon housing, optical fiber, and both polymer and ceramic adhesives is presented. The fabrication of the sensor is scalable using a batch process for the diamond diaphragm and sensor housing structure and an automated fiber insertion and mounting process. The polymer adhesive renders a good airtight sealing of the cavity, and the ceramic adhesive ensures good linearity of pressure sensing in the temperature range up to and 65 °C, which is the highest temperature at which pressure calibration was performed. With the added ceramic adhesive, temperature measurement of the sensor could be performed up to 275 °C without significant signal degradation. The experimental study shows good linearity and high sensitivity from the pressure calibration. The proposed sensor is expected to benefit various fields such as biomedical sensing and industrial sensing by providing low-cost and high-accuracy sensors with excellent chemical resistance and good temperature resistance.
